# Clinical, Radiological, and Genetic Profile of Patients with *FA2H*-Associated Neurodegeneration: Eight Cases from India and a Review of the Literature

**DOI:** 10.5334/tohm.1162

**Published:** 2026-03-05

**Authors:** Vikram V. Holla, Riyanka Kumari, Neeharika Sriram, Nitish Kamble, Jitender Saini, Ravi Yadav, Babylakshmi Muthusamy, Pramod Kumar Pal

**Affiliations:** 1 Department of Neurology, National Institute of Mental Health and Neurosciences, Hosur Road, Bangalore-560029, India; 2 Institute of Bioinformatics, International Technology Park, Bangalore-560066, India; 3 Manipal Academy of Higher Education, Manipal-576104, Karnataka, India; 4 Department of Neuroimaging and Interventional Radiology, National Institute of Mental Health and Neurosciences, Hosur Road, Bangalore-560029, India

**Keywords:** Dystonia, heme-binding, SPG35, FA2H, FAHN, NBIA

## Abstract

**Background:**

Autosomal recessive spastic paraplegia 35 (SPG35), also known as Fatty acid hydroxylase-associated neurodegeneration (FAHN), is a rare recessive neurodegenerative disorder with or without ataxia, dystonia, and other neurological findings. It is caused by genetic variants in *FA2H*, which encodes fatty acid 2-hydroxylase.

**Objective:**

To report the clinical, electrophysiological, radiological, and genetic profile of patients diagnosed with FAHN.

**Methods:**

We performed a retrospective chart review of genetically proven cases of FAHN from our database.

**Results:**

We identified eight patients (6 females) with genetically proven FAHN. All patients presented with first-decade onset pyramidal syndrome with or without ataxia and with radiological findings of callosal atrophy, peri-ventricular white matter hyperintensity, and cerebellar atrophy. Iron accumulation was observed in four of them. Whole exome sequencing revealed seven unique variants including three missense variants (c.83G>C;p.Arg28Pro, c.130C>A;p.Pro44Thr, and c.703C>T;p.Arg235Cys), a stop-gain variant (c.379C>T;p.Arg127Ter), a frameshift deletion variant (c.536delT;p.Leu179Argfs*62), a in-frame deletion variant (c.200_202del;p.His67del) and a in-frame duplication variant (c.86_97dup;p.Arg29_Arg32dup). The variants p.Pro44Thr, p.Arg28Pro, p.Arg29_Arg32dup, and p.His67del are located in the iron-binding region, and the p.Arg235Cys in the hydroxylase domain. The other two variants, p.Arg127Ter and p.Leu179Argfs*62, predictively cause protein truncation, leading to loss of the transmembrane domain and the fatty acid hydroxylase domain, which in turn may result in disruption of fatty acid alpha-hydroxylase activity of FA2H.

**Conclusion:**

Our study identifies novel variants associated with FA2H in FAHN patients, highlighting their possible roles in iron binding and in the loss of the transmembrane and catalytic domains.

Fatty Acid Hydroxylase Associated Neurodegeneration (FAHN) is a rare autosomal recessive neurodegenerative disorder characterized by a deficiency in the enzyme fatty acid 2-hydroxylase (FA2H), encoded by the *FA2H* located on chromosome 16q23 (OMIM #611026) [1,2]. It belongs to a subset of neurodegenerative disorders known as Neurodegeneration with Brain Iron Accumulation (NBIA), distinguished by the accumulation of iron in the basal ganglia region of the brain [3]. Three overlapping phenotypes have been described with *FA2H* genetic abnormality: leukodystrophy with spasticity and dystonia; fatty acid hydroxylase-associated neurodegeneration; and hereditary spastic paraplegia (SPG) type 35 (SPG35) [4]. The clinical manifestation of homozygous or compound heterozygous variants associated with *FA2H* includes childhood onset of spasticity, ataxia, dystonia, dysarthria, leukodystrophy, and mild cognitive impairment [3,5]. Additionally, various other neurological manifestations, such as optic atrophy and seizures, may manifest with varying degrees of severity [6,7]. *Rattey et. al*. 2019 characterized the MRI hallmark for FAHN as an acronym “WHAT”: white matter changes, hypo-intensity of the globus pallidus, ponto-cerebellar atrophy, and thin corpus callosum. They also reported unusually bristly hair in FAHN patients [8].

*FA2H* encodes fatty acid 2-hydroxylase, a 372-amino-acid enzyme that hydroxylates straight fatty acids at the C-2 position, an essential step in the production of 2-hydroxy glycosphingolipids (GSLs), which are vital building blocks of myelin [2,7]. The FA2H enzyme is an NAD(P)H-dependent monooxygenase that belongs to the fatty acid hydroxylase/desaturase gene family and localises to the endoplasmic reticulum [9]. It is the sole enzyme identified in eukaryotes capable of hydroxylating linear fatty acids at the C-2 position, with the brain exhibiting the highest level of *FA2H* expression. Following the brain, the colon also demonstrates notable expression, whereas the testis, prostate, pancreas, and kidney exhibit comparatively lower levels of expression [2]. FA2H protein contains an N-terminal Cytochrome b5 domain (8–86 amino acids) and a hydroxylase domain (219–361 amino acids). Variants located in these domains have been reported to cause loss of function of the enzyme [2].

The pathophysiological mechanisms underlying FAHN are multifaceted, involving disruptions in myelin homeostasis, aberrant iron metabolism, and mitochondrial dysfunction, but the exact aetiology remains elusive [10]. Variants in the *FA2H* have been implicated, leading to impaired FA2H enzymatic activity and subsequent dysregulation of lipid metabolism within the CNS. This dysregulation culminates in myelin instability, neuronal dysfunction, and ultimately leads to neurodegeneration. Additionally, defective endosomal recycling, autophagy, and mitochondrial morphology also lead to the diseased condition [10,11]. Recently, lipidomic profiling of blood from patients with the neurodegenerative condition spastic paraplegia-35 (SPG35) showed that reduced enzyme activity disrupts sphingolipid and GSL synthesis pathways. Disrupted lipid metabolism may induce apoptosis in oligodendrocytes and Schwann cells, crucial for myelin maintenance, potentially causing demyelination and subsequent brain degeneration [12]. This study identifies variants in *FA2H* in patients presenting with signs of demyelination and neurological movement disorder symptoms, which aligns with previous claims that dysfunction of FA2H can lead to myelination abnormalities, impaired axonal function, and loss of the myelin sheath as observed in knockout mice [13]. In this study, we present the clinical, radiological, electrophysiological, and genetic observations from eight patients of FAHN of Indian ethnicity.

## Methods

In this retrospective study conducted at a tertiary neurology referral centre in India, we screened our database for patients with genetically confirmed FAHN. The available clinical, radiological, electrophysiological, and genetic data were documented and analysed. The data were analysed using descriptive statistics and presented as medians (ranges) or frequencies and percentages. The study was approved by the National Institute of Mental Health and Neurosciences Institute Ethics Committee, and written informed consent was obtained from the patients for participation in this study, video recording, and publication in print or online. In addition, we reviewed the literature on PubMed using the keyword “FA2H” for previously reported bi-allelic FAHN cases with clinical details published in English. We extracted the clinical, radiological, and genetic details from these reports and tabulated them.

## Results

We identified eight patients (6 females) with genetically proven FAHN, with a median age at onset of 4.5 years (4–10 years) and a median duration of illness of 3.5 years (1–19 years). The clinical, imaging, electrophysiological, and genetic details are summarized in Table 1. The MRI findings of seven patients are provided in Figure 1, and the clinical examination videos of five patients are provided as supplementary videos e1 to e5.

**Table 1:** Clinical, electrophysiological, radiological, and genetic findings of patients with FA2H-related neurodegeneration in our cohort.


VARIABLES	CASE-1	CASE-2	CASE-3	CASE-4	CASE-5	CASE-6	CASE-7	CASE-8

Demographics and history								

Age/AAO/Gender	10y/7y/F	29y/10y/M	8y/4y/F	10y/4y/F	7y/5y/F	8y/7y/F	15y/5y/F	7y/6y/F

Consanguinity/FH	Yes/No	Yes/No	Yes/No	Yes/No	Yes/No	No/Yes	Yes/No	No/No

DD	No	No	No	Mild – Motor	Mild – motor	No	No	

Presenting symptom	Difficulty walking, and speaking	Difficulty walking, speaking, and poor scholastic performance	Difficulty walking and speaking	Difficulty walking and speaking, and hand incoordination	Difficulty walking	Difficulty walking	Difficulty walking, speaking, and writing difficulty	Difficulty walking, speaking, and hand incoordination

*Examination*								

Cognition	Normal	Impaired	Impaired	Normal	Normal	Normal	Impaired	Normal

Eye movements	Saccadic pursuits, hypermetric saccade	Slow saccades, broken pursuit, exotropia	Normal	Upgaze restriction,	Normal	GEN	GEN	GEN

Fundus	Mild temporal pallor	Normal	Normal	Normal	Mild temporal pallor	Normal	Normal	Normal

Speech	Spastic-ataxic	Spastic	Ataxic	Spastic-Ataxic	Spastic	Normal	Spastic	Spastic-Ataxic

Tone	Spastic in all 4 limbs	Spastic in all 4 limbs	Spastic in all 4 limbs	Spastic in legs	Spastic in legs	Spastic in all 4 limbs	Spastic in all 4 limbs	Spastic in all 4 limbs

Power	Normal	Normal	Normal	Normal	Normal	Normal	Lower limb	Normal

DTR	Brisk in all 4 limbs	Brisk in all 4 limbs	Brisk in all 4 limbs	Brisk in all 4 limbs	Brisk in all 4 limbs	Brisk in all 4 limbs	Brisk in all 4 limbs	Brisk in all 4 limbs

Plantar	Extensor bilaterally	Extensor bilaterally	Extensor bilaterally	Extensor bilaterally	Extensor bilaterally	Extensor bilaterally	Extensor bilaterally	Extensor bilaterally

Cerebellar	Ataxia	Normal	Ataxia	Ataxia	Normal	Normal	Ataxia	Ataxia

Gait	Spastic ataxic	Wheelchair bound	Spastic ataxic	Spastic ataxia	Spastic	Spastic	Spastic-ataxic	Spastic-ataxic

Dystonia	Distal limbs	None	None	None	None	None	None	None

Ambulation	Without support	Wheelchair bound	Without support	With support	With support	Without support	With support	Without support

Pes-cavus	Present	Present	Absent	Absent	Absent	Absent	Absent	Absent

Contracture	Ankle	Hamstring, ankle	Ankle	Ankle	None	None	Ankle	Absent

*Investigations*								

Blood investigations#	Normal	Normal	Normal	Normal	Normal	Normal	Normal	Normal

NCS	Normal	Sensory axonal	NA	NA	Normal	NA	NA	Normal

VEP	R-126 µs/L-132 µs	NA	NA	L-133.8 µs/R-127.2 µs	NA	NA	NA	Normal

BAER	Normal	NA	NA	Normal	Normal	NA	NA	Normal

SSEP	Normal	Normal	NA	Normal	Normal	NA	NA	Normal

*MRI Brain*								

Callosal atrophy	Present	Present	Present	Present	Present	Present	Present	Present

Optic atrophy	Present	Present	Absent	Present	Present	Absent	Absent	Absent

Cerebellar atrophy	Present	Present	Present	Present	Present	Present	Present	Present

PVWMH	Present	Present	Present	Present	Present	Present	Present	Present

GPi hypointensity	Present (T2 & SWI)	Present (T2 & SWI)	Absent	Present (T2 & SWI)	Present (T2)	NA	NA	Present (T2)

*Variants identified in the FA2H (ENST00000219368)*								

Zygosity	Homozygous	Homozygous	Homozygous	Homozygous	Homozygous	Homozygous	Homozygous	Homozygous

Exon	Exon-1	Exon-1	Exon-4	Exon-1	Exon-3	Exon-5	Exon-1	Exon-1

Nucleotide change	c.200_202del	c.130C>A	c.536delT	c.83G>C	c.379C>T	c.703C>T	c.200_202del	c.86_97dup

Amino acid change	p.His67del	p.Pro44Thr	p.Leu179ArgfsTer62	p.Arg28Pro	p.Arg127Ter	p.Arg235Cys	p.His67del	p.Arg29_Arg32dup

Consequence	In-frame deletion	Missense	Frameshift, truncation	Missense	Nonsense	Missense	In-frame deletion	In-frame duplication

ACMG classification	Likely pathogenic	Likely pathogenic	Pathogenic	Likely pathogenic	Pathogenic	Likely pathogenic	Likely pathogenic	Likely Pathogenic

	PM_1,2,4_,PP_3_	PM_1,2,5_,PP_2,3_	PVS_1_,PM_2_,PP_3_	PM_1,2_,PP_2,3_	PVS_1_,PM_2_,PP_3,5_	PM_2,3_,PP_3,4,5_	PM_1,2,4_,PP_3_	PM_1,2,4_,PP_4_

Novel variant	Yes	Yes	Yes	Yes	No	No	Yes	Yes

CADD	NA	32	NA	30	NA	32	NA	NA

REVEL	NA	0.9	NA	0.66	NA	0.85	NA	NA


#: Blood investigations contain complete blood count, renal function test, liver function test, thyroid function test, vitamin B12, homocysteine, and tandem Mass spectroscopy. AAO: Age at onset; BAER: Brainstem auditory evoked potential; CADD: Combined Annotation Dependent Depletion; DTR: Deep tendon reflexes; F: Female; FH: Family history; GEN: Gaze-evoked nystagmus; GPi: Globus pallidi interna; M: Male; MRI: Magnetic resonance imaging; NA: Not available; NCS: Nerve conduction study; PVWMH: Peri-ventricular white matter hyperintensity; REVEL: Rare Exome Variant Ensemble Learner; SSEP: Somatosensory evoked potential; SWI: Susceptibility weighted imaging; VEP: Visual evoked potentials; y: years.

**Figure 1:**
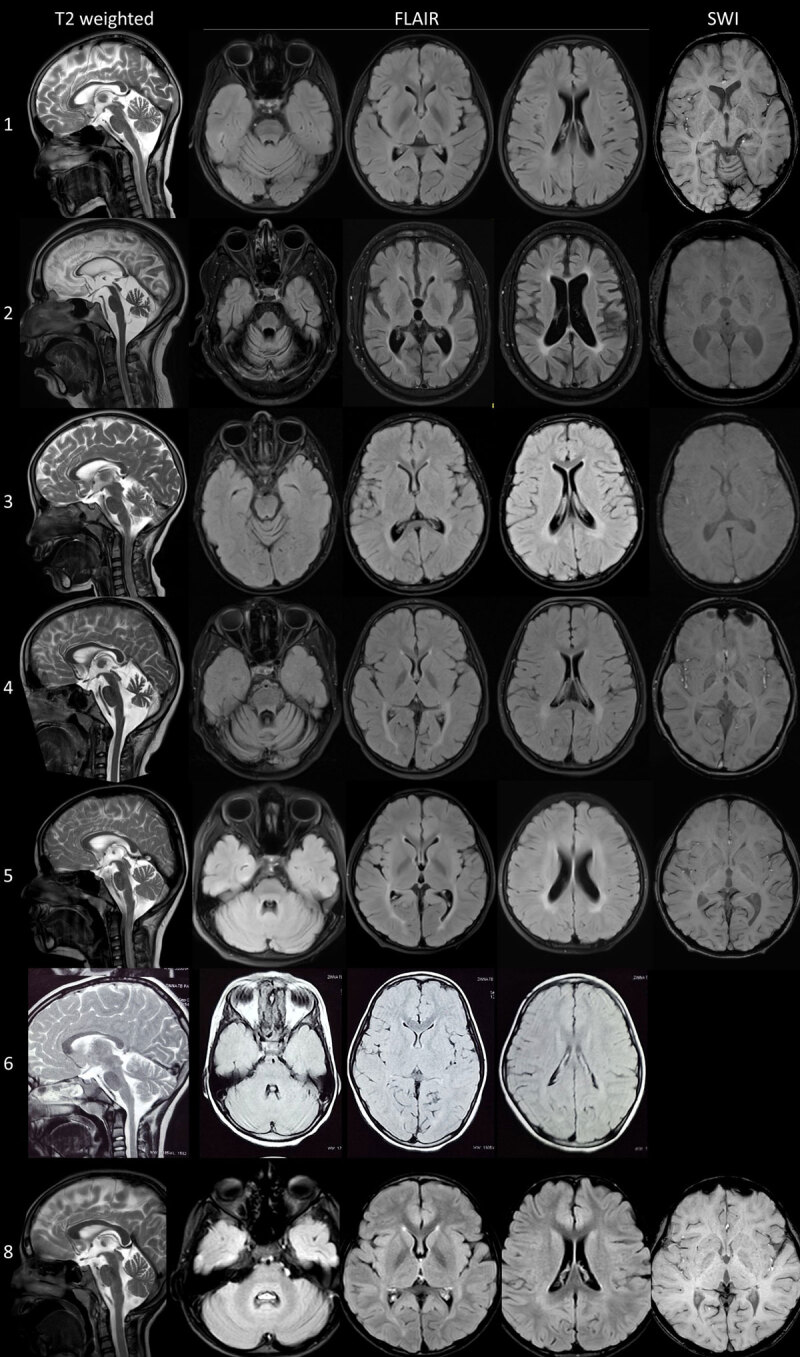
Magnetic resonance imaging of the cohort. T2, FLAIR, and SWI sequences of the MRI brain of the patients demonstrating callosal atrophy, cerebellar atrophy, and periventricular white matter hyperintensity in all, GPi hypointensity on T2 sequences in Cases 1, 2, 4, 5, and 8, and on SWI sequences in Cases 1, 2, and 4. Snapshots of the MRI of Case 7 were not available. FLAIR: Fluid attenuated inversion recovery; GPi: Globus pallidi interna; MRI: Magnetic resonance imaging; SWI: Susceptibility weighted imaging.

### Clinical details

Walking difficulty (8/8) was the most common presenting symptom. Patients also experienced speech slurring (6/8), upper-limb clumsiness (3/8), cognitive symptoms (2/8), and mild motor global developmental delay (2/8). A positive family history of similar clinical findings and consanguineous parentage was noted in 1 and 6 patients, respectively.

On examination, all patients had lower-limb-predominant pyramidal involvement, including spasticity, hyperreflexia, and extensor plantar responses. In addition, cerebellar signs were present in five patients, all of whom had lower-limb and gait-predominant ataxia; four also had upper-limb and speech involvement, and three had eye involvement in the form of hypermetric saccades or nystagmus. Cognition was impaired in two patients, mild optic atrophy in two patients, oculomotor abnormalities in six patients (nystagmus: 3, broken pursuit: 2, upgaze restriction: 1, exotropia: 1), dysarthria in seven patients, foot weakness in one patient, and a normal sensory examination in all. Four patients were able to walk without support, three with support, and one was wheelchair dependent. Among the seven ambulant patients, spastic-ataxic gait was noted in five and pure spastic gait in two. Additionally, one patient had hand dystonia, two had pes cavus, and five had ankle contracture.

### Electrophysiology and imaging

Electrophysiological evaluation revealed sensory axonal neuropathy (1/4, others 3 normal), prolonged p100 latency on visual evoked potential (VEP) (2/3, others were normal), normal brainstem auditory evoked potentials (BAER) (4/4), and normal lower limb somatosensory evoked potentials (SSEP) (5/5). MRI brain was available in all eight patients and showed callosal atrophy (8/8), cerebellar atrophy (8/8), periventricular white matter T2 and fluid attenuated inversion sequence (FLAIR) hyperintensity (8/8), hypointensity of globus pallidus interna (GPi) on T2/FLAIR (4/6), and optic atrophy (4/8) (Figure 1 and Table 1).

### Genetic testing

**Figure 2:**
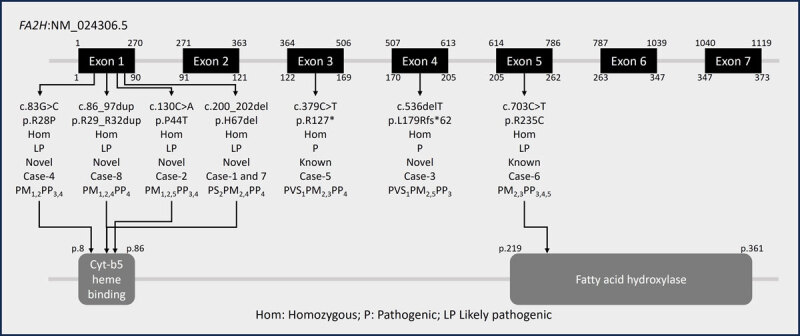
Cartoon depicting the details and location of the variants identified in the cohort on the *FA2H*.

*FA2H* has two isoforms, and the canonical isoform (NM_024306.5) encodes a 372-amino acid protein that has a Cytochrome b5 heme-binding domain (8–86), a transmembrane domain (168–188), and a fatty acid hydroxylase domain (219–361) (Figure 2). Exome sequencing was performed in all eight patients, revealing seven unique pathogenic or likely pathogenic variants in *FA2H*, all in a homozygous state. None of the patients carried any additional homozygous variant relevant to the phenotype. Of the seven variants, two variants have been previously described (c.703C>T;p.Arg235Cys and c.379C>T;p.Arg127Ter) (4), and the other five variants were novel (c.200_202del;p.His67del, c.130C>A;p.Pro44Thr, c.536delT;p.Leu179ArgfsTer62, c.83G>C;p.Arg28Pro, c.86_97dup;p.Arg29_Arg32dup) (Figure 2). Missense variants were the most frequent (3 variants). In addition, there was one variant each of in-frame deletion (c.200_202del;p.His67del), in-frame duplication (c.86_97dup;p.Arg29_Arg32dup), stop-gain (c.379C>T;p.Arg127Ter), and frameshift deletion (c.536delT;p.Leu179ArgfsTer62). One novel variant (c.200_202del;p.His67del) was seen in two unrelated patients.

Patient-1 and Patient-7, both born to consanguineous parents, were identified with a 3-bp deletion within exon 1 of the *FA2H* (NC_000016.10:g.74774554_74774556del;c.200_202del;p.His67del), leading to an in-frame deletion of the histidine amino acid at the 67^th^ position. The variant is rare, observed in 0.0007% in gnomAD v4.0 Genomes and 0.001% in the in-house database, with no homozygous variants reported. The deleted sequence TGT is highly conserved, with a PhyloP100 score of 7.07 for this variant. The deletion of histidine at the 67^th^ codon is expected to shorten the Cyt-b5 heme-binding domain, thereby altering its structure. The Sanger sequencing in Case-1 showed this variant as homozygous in the patient and heterozygous in the unaffected parents. Sanger sequencing could not be performed in Patient-7.

Patient-2, born to consanguineous parents, was identified with a homozygous missense variant in exon-1 of the *FA2H* (NC_000016.10:g.74774626G>T;c.130C>A;p.Pro44Thr). The variant is rare, observed in 0.00007% in gnomAD v4.0 Exomes and not reported in the in-house database, with no homozygous variants reported. The PhyloP conservation score for this variant is 5.65; SIFT and Polyphen2 scores predict it as deleterious. The variant has a Combined Annotation Dependent Depletion (CADD) score of 32 and a Rare Exome Variant Ensemble Learner (REVEL) score of 0.9. It is located adjacent to the heme binding residue His43. Previously, a cytosine-to-thymine variant resulting in the same p.Pro44Ser is reported to be associated with *FA2H-*related HSP conditions in Turkish and Czech patients [6,14]. Sanger sequencing confirmed the variant as homozygous in the patient, heterozygous in the unaffected parents, and absent in the unaffected sibling.

Patient-3, born to consanguineous parents, was identified with a novel homozygous 1-bp deletion in the exon 4 of the FA2H (NC_000016.10:g.74726302del;c.536delT;p Leu179Argfs*62) that predictively results in a frameshift and premature truncation of the protein. The variant is rare, observed in 0.00007% in gnomAD v4.0 Exomes and not reported in the in-house database, with no homozygous variants reported. The resulting frameshift introduces a premature termination codon 62 codon downstream of 179 codon, is located in exon 4 of 7, is neither in the last exon nor near the start codon, and is positioned more than 50–55 nucleotides upstream of the final exon–exon junction. Accordingly, it does not meet established nonsense-mediated decay escape criteria, and in-silico tools (aenmd and MutationTaster) predict nonsense-mediated mRNA decay. The premature truncation of the protein results in the loss of transmembrane domains and zinc-binding sites. Sanger sequencing revealed that this variant was homozygous in the patient and heterozygous in the mother. The father’s sample was not available for the Sanger sequencing.

Patient-4, born to consanguineous parents, was identified with a novel homozygous missense variant in exon-1 of the *FA2H* (NC_000016.10:g.74774673C>G;c.83G>C;p.Arg28Pro). This variant is neither reported in the gnomAD database nor in the in-house database. The variant is predicted to be deleterious by Polyphen and MutationTaster and is located in the Cytochrome b5 heme-binding domain. The variant has a CADD score of 30 and a REVEL score of 0.66. Sanger sequencing confirmed that this variant was homozygous in the patient and heterozygous in the mother; the father’s sample was unavailable for segregation analysis (Figure 2).

Patient-5, born to consanguineous parents, was identified with a homozygous nonsense variant in the exon-3 of the *FA2H* (NC_000016.10:g.74727371G>A;c.379C>T;p.Arg127*). The variant is rare, observed in 0.0006% in gnomAD v4.0 and not reported in the in-house database, with no homozygous variants reported. It is reported as pathogenic in ClinVar (rs1210045384) and causes a stop-gain mutation, resulting in a premature protein truncation and loss of transmembrane domains and zinc-binding sites. The resulting premature termination codon is located in exon 3 of 7, is neither in the last exon nor near the start codon, and is positioned more than 50–55 nucleotides upstream of the final exon–exon junction. Accordingly, it does not meet established nonsense-mediated decay escape criteria, and in-silico tools (aenmd and MutationTaster) predict nonsense-mediated mRNA decay. Sanger sequencing showed this variant as homozygous in the patient and heterozygous in the unaffected parents.

Patient-6, born to non-consanguineous parents, was identified with a homozygous missense variant in exon 5 of the *FA2H* (NC_000016.10:g.74719071G>A;c.703C>T;p.Arg235Cys). The variant is rare, observed in 0.0009% in gnomAD with no homozygous variants reported. It is reported as pathogenic in ClinVar (rs387907039) and has been previously reported [4]. The PhyloP conservation score for this variant is 8.79; SIFT and Polyphen2 scores predict it as deleterious, has a CADD score of 32 and a REVEL score of 0.85. Although the parents were reportedly unrelated, the possibility of distant unreported consanguinity cannot be excluded, given the patient’s homozygosity for the variant and a positive family history in the brother. We could not perform Sanger sequencing in the parents and the brother.

Patient-8, born to non-consanguineous parents, was identified with a homozygous in-frame duplication variant in the exon 1 of the *FA2H* (NC_000016.10:g.74774658A>AGGCGGGCCCCGC;c.86_97dup;p.Arg29_Arg32dup). The variant is rare, observed in 0.0007% in gnomAD v4.0 Genomes, in a heterozygous state, and absent in the in-house database. It is expected to lengthen the protein in the Cyt-b5 heme-binding domain, thereby altering the protein’s structure. Sanger sequencing confirmed the variant as homozygous in the patient and heterozygous in the unaffected parents. Although the parents were reportedly unrelated, the possibility of unreported consanguinity cannot be excluded, given that the variant is homozygous in the patient.

### Review of literature

On searching PubMed with the keyword “FA2H”, we identified a total of 209 articles as of 12-05-2025. Among them, 43 articles described patients with FAHN, including clinical, radiological, and/or genetic details. Two reports duplicated the same patient [15,16]. Hence, we were left with 42 articles comprising 130 patients. Including the current study, there are a total of 138 cases.

#### Clinical features

Less than 10% of the total cohort had age at onset after the first decade (11/138). Gender distribution was almost equal (71/138). The majority were from Asia and the Middle East region (79/138). Global developmental delay was noted in 7/138 (5%). At least one sign of pyramidal involvement in the form of spastic dysarthria, lower limb predominant spasticity, brisk deep tendon reflexes, and/or extensor plantar response was noted in all the patients where the details were available. In addition, ataxia and dystonia were frequent extrapyramidal signs observed. Additional notable neurological findings were optic atrophy, saccadic abnormalities, nystagmus, upgaze restriction, strabismus, dysphagia, lower limb weakness, pes cavus, contracture, bowel and bladder abnormalities, seizures, and cognitive impairment in a variable proportion of patients.

#### Investigations

At least some MRI details were available for 100/138 patients. Periventricular white matter abnormalities were the most frequent (80/92, 87%), followed by cerebellar atrophy (71/90, 78.9%) and thinning of the corpus callosum (57/86, 66.3%). Globus pallidus hypointensity was observed in 35/53 patients (66%). Notably, iron deposition or mineralization was mentioned only in 19/49 patients (38.8%).

Nerve conduction study and/or electromyography were abnormal in a quarter of patients (13/53, 24.5%), with over two-thirds having sensory-motor axonal neuropathy (9/13, 69.2%) and the remaining having sensory axonal neuropathy (4/13, 30.8%). Prolonged or absent central motor conduction time on motor evoked potential was seen in three-fourths of the cases (9/12, 75%). Among sensory evoked potentials, VEP was most frequently abnormal (10/17, 58.8%), followed by SSEP (5/19, 26.3%) and BAER (1/7, 14.3%).

#### Variant details

In total, variant details of 92 families (138 patients) are available. Of these, in 67 families the variants were homozygous, and in 24 families, compound heterozygous. In one family, only a single heterozygous variant was identified with an otherwise classical phenotype [17]. With respect to the variant combination, in 47 families, there were bi-allelic missense variants, in 24 families, there were biallelic truncating variants (combination of splice site, stop-gain, frameshift, and/or copy number loss), in 9 families, a missense-truncating combination was noted, and in the remaining 12 families, bi-allelic in-frame variants were noted.

Seventy-nine unique variants have been reported so far in the literature. Of these, the majority are missense variants (49/79, 62%), followed by truncating variants (Stop-gain: 10/79, 12.7%; frameshift: 9/79, 11.4%; splice-site: 4/79, 5.1%; CNV: 3/79, 3.8%) and in-frame deletion/duplication variants (4/79, 5/1%). Among the missense variants, 16/49 variants were in the cyt-b5 domain, and 22/49 variants were in the fatty-acid hydroxylase domain.

## Discussion

In this investigation, we present eight cases of FAHN from eight distinct families, with six patients having consanguineous parentage and one patient having a positive family history in a sibling. All the patients exhibited first-decade onset chronic progressive lower-limb predominant spastic paraparesis with or without cerebellar ataxia, cognitive impairment, optic atrophy, oculomotor abnormality, dystonia, and pes cavus. Electrophysiologically, sensory axonal neuropathy and prolonged p100 latencies were observed in 1 and 2 patients, respectively, while brainstem auditory and somatosensory evoked responses were normal in all those tested. All patients had callosal and cerebellar atrophy and periventricular white matter hyperintensities. GPi mineralization was noted only in four patients. Exome sequencing revealed homozygous variants in *FA2H* in all patients, of which four were novel.

### Clinical features

As per the available literature, the majority of patients have a first-decade onset (Supplementary Table-1) [8]. Global developmental delay has been reported, but is observed in a minority of patients. Conversely, cognitive impairment appears to be common, with severity ranging from mild to severe impairment. Additional psychiatric and behavioural abnormalities have also been reported rarely. Eye abnormalities, including optic atrophy, strabismus, ophthalmoplegia, nystagmus, saccadic abnormalities, and oculomotor apraxia, have been reported in multiple cases. However, owing to missing data in the majority, the actual prevalence of these in FAHN is difficult to comment upon. Dysarthria is almost universal, but its character is not reported uniformly; it appears spastic in the majority. Additional dysphagia was observed in a minority of cases. There were no reports of any pseudobulbar effect.

Based on the literature review, pyramidal signs appear to be a sine qua non feature in patients with FAHN. It can be in the form of spasticity, brisk deep tendon reflexes, and/or extensor plantar response. The spasticity and hyperreflexia are often present predominantly in the lower limb, and hence, FAHN is classified under the SPG group as SPG35 [4]. In addition to pyramidal signs, additional movement disorder phenomenologies are not infrequent, with ataxia being the most common, followed by dystonia. Both ataxia and dystonia tend to be lower-limb predominant and symmetrical. Rigidity, bradykinesia, rest tremor, and myoclonus have also been rarely reported [8].

### Imaging

Radiologically, all patients had callosal and cerebellar atrophy and periventricular white matter hyperintensity, while mineralization was demonstrated in only half of the cases (Supplementary Table-2) [8,18]. In a separate investigation conducted in India, Sait et al. documented eight cases of FAHN, all of which manifested during childhood, with an average onset age of five years. Consistent with our observations, the MRI findings in all these patients showed periventricular white matter involvement in the bilateral parietal regions. Additionally, varying extents of brainstem and cerebellar atrophy, along with corpus callosum thinning, were consistently observed across all individuals. However, only one patient in their cohort showed iron accumulation in the brain, whereas iron accumulation is observed in half of our cases [19].

### Electrophysiology

Electrophysiologically, motor evoked conduction time appears to be the most sensitive test, with three-fourths of the tested showing abnormality, followed by visual pathway impairment in more than half of the patients. Peripheral nerve abnormality is observed in approximately one-fourth of cases, with all cases showing sensory impairment, with or without motor abnormality. The electrophysiological findings are in line with those observed in cases of HSP except for a prolonged VEP. Visual pathway abnormalities, including a pale optic disc, MRI optic nerve atrophy and hyperintensity, and prolonged or absent VEPs, may help differentiate HSP due to FA2H from other HSPs.

### Genetic details

Genetic analysis of all eight families identified three missense variants (c.130C>A; p.Pro44Thr, c.83G>C;.Arg28Pro, and c.703C>T; p.Arg235Cys), one in frame deletion (c.200_202del; p.His67del), one frameshift variant (c.536delT;p.Leu179Argfs*62), one stop-gain variant (c.379C>T;p.Arg127Ter) and one in-frame duplication variant (c.86_97dup; p.Arg29_Arg32dup). Variants c.200_202del, c.130C>A, c.83G>C, and c.86_97dup identified in Case-1, Case-7, Case-2, Case-4, and Case-8 are situated within exon 1, which encompasses the cytochrome b5-heme binding domain responsible for intramolecular electron transport during catalytic activity [2]. The variant c.200_202del exhibits an in-frame deletion of a histidine residue at the 67^th^ position, which is directly associated with the heme group. This variant was identified in two unrelated patients in our study. Case 2 had a novel missense variant c.130C>A; p.Pro44Thr. Previously, a variant with a different nucleotide change at the same loci (c.130C>T) that results in a different amino acid substitution at the same codon (p.Pro44Ser) has been reported in a Turkish and a Czech male associated with the disease conditions [6,14]. Additionally, another variant, c.131C>A, which results in an amino acid substitution at codon 44 (p.Pro44Gln), has also been documented as disease-causing, and, interestingly, it was reported to be caused by uniparental disomy [20]. These variants are located adjacent to the heme-binding site at codon 43, which encodes histidine. The novel missense variant c.83G>C; p.Arg28Pro that substitutes the conserved arginine residue at the 28^th^ position and the in-frame duplication variant c.86_97dup; p.Arg29_Arg32dup that extends the protein by 4 amino acids are both situated within the cytochrome b5-heme binding region. These variants are predicted to affect the function of the protein’s cytochrome b5 domain. The importance of the heme-binding domain is supported by a comparative lipidomic analysis of the patient and control groups, which reveals notable differences in lipid metabolites resulting from the deletion of the heme-binding domain of FA2H. In our study, three out of five variants lie in the first exon, specifically in the Cytochrome b5-heme binding domain, which is important for the catalytic activity of the FA2H enzyme [2,12].

Case 3 and Case 5 present with truncating variants c.536delT; p.Leu179ArgfsTer62 and c.379C>T; p.Arg127Ter, respectively. Deletion of T at position 536 is anticipated to induce a frameshift variation in the codon, resulting in premature termination of the protein with only 241 amino acids, consequently leading to the loss of the fatty acid hydroxylase domain. In Case 5, the variant c. 379C>T introduces a premature stop codon UGA at 127^th^ position, resulting in the loss of the hydroxylase domain. In Case 6, a previously known variant was identified at c.703C>T, resulting in an amino acid substitution at the 235^th^ position. This position lies between the histidine-containing zinc binding sites 234 and 239 within the fatty acid hydroxylase domain. The myelin sheath is crucial for the transmission of electrical signals in both the central and peripheral nervous systems. FA2H is the sole enzyme identified in eukaryotes capable of hydroxylating linear fatty acids at the C-2 position, a step important for myelin synthesis. Variants in *FA2H* are associated with hereditary leukodystrophy and spastic paraparesis in humans, underscoring its important role in the nervous system [2,21]. Alterations in these amino acids caused by the reported variants are predicted to impair catalytic activity, leading to disruptions in lipid metabolism and potentially contributing to the development of neurological disorders such as FAHN.

FAHN is a relatively rapidly progressive SPG type with loss of ambulation noted over 5–10 years of disease progression. Therapeutically, symptomatic treatment and rehabilitative measures remain the mainstay, with no studies on disease-modifying therapy. The chelation therapy tried in PKAN has not been studied in FAHN patients. Symptomatic therapy in the form of baclofen or other anti-spastic medications for pyramidal signs, botulinum toxin injections for spasticity, regular physiotherapy and monitoring for visual dysfunction is advised for patients with FAHN. Genetic counselling is important to make the parents aware of the risk of the disease in the subsequent pregnancy and options to prevent it.

This study has several limitations. Owing to its retrospective design, complete investigative data were not available for all patients. Sanger sequencing to confirm segregation of the identified variants could not be performed in some parents and extended family members. Additionally, functional studies to validate the pathogenicity of the novel variants identified in our cohort could not be undertaken.

In conclusion, we present the clinical and genetic profiles of eight patients from unrelated families, and report five novel variants in *FA2H*. FAHN should be considered in the first decade onset of chronic progressive SPG syndromes, either isolated or with associated ataxia, optic atrophy, neuropathy, dystonia and seizures. Although classified as an NBIA, iron deposition is not universal. Our results highlight the importance of genetic and radiological investigations in the diagnosis and management of FAHN. Leukodystrophy in young children without any detectable biochemical abnormality should be considered for FAHN diagnosis.

## Data Accessibility Statement

The data is available to the corresponding author upon a reasonable request.

## Additional Files

The additional files for this article can be found as follows:

10.5334/tohm.1162.s1 Supplementary Table 1. Clinical features associated with FA2H variants in the reported patients.

10.5334/tohm.1162.s2 Supplementary Table 2. MRI features and FA2H variant details in the reported patients.

10.5334/tohm.1162.s3 Supplementary video-e1. **Video of Case-1.** The video demonstrates saccadic dysmetria and saccadic pursuits, mild bilateral hand dystonia, dysdiadokokinesia, impaired finger-nose test and knee-heel-shin test, and spastic ataxic gait.

10.5334/tohm.1162.s4 Supplementary video-e2. **Video of Case-4.** The video demonstrates spastic-ataxic dysarthria, impaired finger-nose test, and spastic ataxic gait. The patient needed support to walk.

10.5334/tohm.1162.s5 Supplementary video-e3. **Video of Case-5.** The video demonstrates spastic dysarthria, impaired finger-nose test, impaired knee-heel-shin test, and spastic gait.

10.5334/tohm.1162.s6 Supplementary video-e4. **Video of Case-6.** The video demonstrates normal speech, normal finger-nose test, knee-heel-shin test, with spastic gait.

10.5334/tohm.1162.s7 Supplementary video-e4. **Video of Case-8.** The video demonstrates spastic-ataxic speech, impaired finger-nose test, and spastic-ataxic gait.
